# Supplementary material for: Empirical methods for controlling false positives and estimating confidence in ChIP-Seq peaks
Source: BMC Bioinformatics. 2008 Dec 5;9:523. doi: 10.1186/1471-2105-9-523 (PMC2628906; doi:10.1186/1471-2105-9-523)
Supplement: Additional File 1 — A variety of html documents from the USeq web site detailing the available applications, their best usage, output file type descriptions, command line menus, etc. [file 1471-2105-9-523-S1.zip › USeqUserGuides/JavaDoc.zip!/JavaDoc/expr/package-tree.html]

expr Class Hierarchy


|  |  |  |
| --- | --- | --- |
| **Overview** \| **Package** \| Class \| Use \| **Tree** \| **Deprecated** \| **Index** \| **Help** | |  |
| **PREV**   **NEXT** | **FRAMES**    **NO FRAMES**     **All Classes** |  |


---


## Hierarchy For Package expr

**Package Hierarchies:**: All Packages

---

## Class Hierarchy

- java.lang.Object
  - expr.**CorrelationMaps**- expr.**CorrelationWindow**- expr.**ExpressedGene** (implements java.lang.Comparable<T>)- expr.**ExpressionScores2CoordinatesViaGFF**- expr.**FileCrossFilter**- expr.**FileMatchJoiner**- expr.**MiRNA**- expr.**MiRNATarget**- expr.**NormalizeAgilentData**- expr.**ParseAgilentData**- expr.**ParseManipulateColumnDataForGeneSifter**- expr.**ParseMiRNATable**- expr.**ParseWormMart**- expr.**StripZeros**

---


|  |  |  |
| --- | --- | --- |
| **Overview** \| **Package** \| Class \| Use \| **Tree** \| **Deprecated** \| **Index** \| **Help** | |  |
| **PREV**   **NEXT** | **FRAMES**    **NO FRAMES**     **All Classes** |  |


---
